# Disease burden and attributable risk factors of lip and oral cavity cancer in China from 1990 to 2021 and its prediction to 2031

**DOI:** 10.3389/fpubh.2024.1419428

**Published:** 2024-09-05

**Authors:** Zhengrong Yu, Xiangming Ma, Hanyu Xiao, Yihong Chen, Yuhang Wu, Jing He, Peiyu Cheng

**Affiliations:** ^1^Department of Stomatology, Zhuhai Hospital of Integrated Traditional Chinese and Western Medicine, Zhuhai, China; ^2^School of Stomatology, Hunan University of Traditional Chinese Medicine, Changsha, China; ^3^School of Medicine, Nankai University, Tianjin, China; ^4^Xiangya Stomatological Hospital, Central South University, Changsha, China; ^5^Department of Epidemiology and Health Statistics, Xiangya School of Public Health, Central South University, Changsha, China; ^6^Xiangtan Central Hospital, Xiangtan, China

**Keywords:** lip and oral cavity cancer, disease burden, prediction, risk factors, China

## Abstract

**Aims:**

This study addresses the essential need for updated information on the burden of lip and oral cavity cancer (LOC) in China for informed healthcare planning. We aim to estimate the temporal trends and the attributable burdens of selected risk factors of LOC in China (1990–2021), and to predict the possible trends (2022–2031).

**Subject and methods:**

Analysis was conducted using data from the Global Burden of Disease study (GBD) 2021, encompassing six key metrics: incidence, mortality, prevalence, disability-adjusted life years (DALYs), years lived with disability (YLDs), and years of life lost (YLLs). Absolute number and age-standardized rates, alongside 95% uncertainty intervals, were computed. Forecasting of disease burden from 2022 to 2031 was performed using an autoregressive integrated moving average (ARIMA) model.

**Results:**

Over the observed period (1990–2021), there were notable increases in the number of deaths (142.2%), incidence (283.7%), prevalence (438.0%), DALYs (109.2%), YLDs (341.2%), and YLLs (105.1%). Age-standardized rates demonstrated notable changes, showing decreases and increases of −5.8, 57.3, 143.7, −8.9%, 85.8%, and − 10.7% in the respective metrics. The substantial majority of LOC burden was observed among individuals aged 40–79 years, and LOC may exhibit a higher burden among males in China. From 2022 to 2031, the age-standardized rate of incidence, prevalence, and YLDs of LOC showed upward trends; while mortality, DALYs, and YLLs showed downward trends, and their estimated values were predicted to change to 2.72, 10.47, 1.11, 1.10, 28.52, and 27.43 per 100,000 in 2031, respectively. Notably, tobacco and high alcohol use emerged as predominant risk factors contributing to the burden of LOC.

**Conclusion:**

Between 1990 and 2021, the disability burden from LOC in China increased, while the death burden decreased, and projections suggest these trends will persist over the next decade. A significant portion of this disease burden to modifiable risk factors, specifically tobacco use and excessive alcohol consumption, predominantly affecting males and individuals aged 40–79 years. Attention to these areas is essential for implementing targeted interventions and reducing the impact of LOC in China.

## Introduction

Cancers pose a significant global health challenge, and are considered a major contributor to disease burden worldwide (1). Lip and oral cavity cancer (LOC) emerge as a substantial component, accounting for an estimated 370,000 new cases and 199,000 deaths globally in 2019 (2). This places LOC at the 16th position both in terms of incidence and mortality rates (3). Within the domain of head and neck malignancies, LOC stands out as the most prevalent. These tumors typically originate at the vermillion border of the lips and extend across the buccal mucosa, encompassing areas such as the tongue, oral cavity, floor, and palate (4). Survival rates underscore the severity of LOC, with only 40–50% of patients surviving beyond 5 years post-diagnosis. Notably, the 5-year survival rate for stage I cancer stands at 80%, plummeting to a mere 20% for advanced stages (stages III/IV) (4–6). It is noteworthy that early-stage LOC commonly presents no discernible symptoms and may be overlooked by non-dental healthcare providers, remaining undiagnosed until the emergence of pain symptoms caused by lesions (7). Given these complexities, assessing the burden of LOC assumes paramount importance in tailoring targeted cancer prevention policies. Such initiatives are indispensable for proactively addressing the formidable challenges posed by cancers.

The Global Burden of Disease (GBD) study conducted a comprehensive assessment and quantification of the disease burden associated with lip and oral cavity cancer (LOC). Recent findings from a study revealed notable disparities in LOC incidence, mortality, and disability-adjusted life years (DALY) rates across the spectrum of socio-demographic indices (SDI) (2). These findings underscore the imperative for countries at varying levels of socioeconomic development to implement tailored measures addressing the substantial burden of LOC within their national contexts. China, with a population comprising approximately one-fifth of the global populace (8), plays a pivotal role in shaping global health outcomes. The repercussions of health developments in China reverberate globally, underscoring the interconnectedness of health outcomes across borders. Moreover, the confluence of rapid demographic aging and heightened exposure to risk factors further complicates the landscape of LOC in China (9, 10). However, despite the significance of these challenges, a paucity of updated comparisons of risk factors and a precise understanding of the disease burden of LOC at the national level in China persists. Addressing these knowledge gaps is essential for informing targeted interventions and policy formulation aimed at mitigating the burden of LOC in China.

This study aims to address the existing research gap by conducting a thorough analysis and making reasonable predictions regarding the disease burden associated with LOC in China. Our analysis will encompass various key indicators, including incidence, prevalence, mortality, disability-adjusted life years (DALYs), years lived with disability (YLDs), and years of life lost (YLLs), alongside an investigation into the corresponding risk factors at the national level. This study is guided by three principal objectives. Firstly, we endeavor to conduct a thorough numerical assessment and trend analysis of LOC in China spanning the previous three decades, with a focus on delineating attained successes and identifying areas ripe for improvement. Secondly, we aim to forecast the potential trajectory of LOC disease burden in China over the ensuing decade. Thirdly, we aspire to furnish critical insights and foundational knowledge essential for mitigating the disease burden of LOC in China. This includes highlighting pertinent risk factors and offering key information to inform prevention and treatment strategies aimed at reducing the prevalence and impact of LOC within the Chinese population.

## Methods

### Overview of GBD 2021 and disease definition

The GBD 2021 provides comprehensive insights into the burden of 371 different health conditions across 204 countries and territories, encompassing a broad spectrum of health data related to disease incidence, prevalence, mortality, and risk factors (11). Data aggregation for the GBD 2021 involved a comprehensive collection process drawing from various sources, including censuses, household surveys, civil registration and vital statistics, disease registries, notifications, health services utilization data, air pollution monitoring, satellite imagery, and other pertinent health-related data repositories (12). Each of these types of data are identified from systematic review of published studies, searches of government and international organization websites, published reports, primary data sources such as the Demographic and Health Surveys, and contributions of datasets by GBD collaborators (12). Each data source that is newly identified and obtained is uniquely tagged by a designated team of librarians and integrated into the Global Health Data Exchange (GHDx). The GHDx ensures public accessibility to the metadata for each source incorporated into the GBD alongside the actual data, contingent upon the approval of the data providers. The GHDx source tool is also available for users to pinpoint the specific datasets utilized for estimating outcomes related to any particular disease or injury in diverse settings. Extensive documentation detailing the data collection methodology and statistical modeling employed in the GBD 2021 is available in peer-reviewed publications (11, 13). In alignment with the International Statistical Classification of Diseases and Related Health Problems, Tenth Revision (ICD-10), LOC was defined using codes C00 to C08 within the GBD 2021 framework. In the context of this specific investigation, the data pertaining to LOC and its associated attributable risk factors spanning the period from 1990 to 2021 were analyzed utilizing the Global Health Data Exchange query tool.^1^

### Measures of disease burden

This study investigated six metrics, comprising incidence, mortality, prevalence, DALYs, YLDs, and YLLs, to elucidate the disease burden associated with LOC. The definitions and methodologies for calculating these indicators have been extensively delineated in prior scholarly works (11–14). In our investigation, we detailed both the point estimates and their respective 95% uncertainty intervals (UI). These intervals were calculated from 1,000 repeated dataset samples, where the upper and lower bounds were established according to the 2.5 and 97.5th percentiles of the uncertainty distribution. Additionally, we performed age-standardized estimates by utilizing the 2021 global age structure (15).

### Attributable risk factors

The GBD 2021 study conducted a comprehensive assessment of the attributable disease burden associated with 88 risk factors and their combinations globally, regionally, and nationally (13). Estimates were generated for both the attributable count and the attributable age-standardized rates of death, DALYs, YLDs, and YLLs across three key categories of risk factors (all risk factors, tobacco, and alcohol use). In line with comparative risk assessment (CRA) methodology, the population attributable fraction (PAF) was computed using the attributable count of specific risk factors. PAF represents the prospective decrease in the current disease burden that could be realized if the overall population exposure to the risk factor had shifted to an alternate or hypothetical risk distribution scenario (16). The quantifiable burden attributable to each factor was determined by multiplying its respective measurement by the PAF (17).

### Statistical analysis

The disease burden associated with LOC was predicted for the period spanning 2022 to 2031 through the application of the autoregressive integrated moving average (ARIMA) model with parameters (*p*, *d*, *q*) denoting the orders of autoregression, degree of differencing, and moving average, respectively. The ARIMA equation is as follows:


$$Y_{t}=\alpha +\emptyset_{1}Y_{t-1}+\emptyset_{2}Y_{t-2}+\ldots +\emptyset_{p}Y_{t-p}+\varepsilon_{t}+\theta_{1}\varepsilon_{t-1}+\ldots +\theta_{q}\varepsilon_{t-q}$$

Where ∅ are θ the autoregressive and moving average parameters, respectively. $Y_{t}$ represents the differenced time series, and $\varepsilon_{t}$ is the value of the random shock at time t. α is a constant. More comprehensive information on the utilization and specifics of this model was furnished in alternative literature sources (18). Initially, if deemed necessary, the raw data underwent logarithmic transformation followed by differential processing on the time series. The series’ stationarity was confirmed through the Augmented Dickey–Fuller (ADF) test, while optimal model parameters (*p* and *q*) were identified utilizing the autocorrelation function (ACF) and partial autocorrelation function (PACF). Selection of the most appropriate ARIMA (*p*, *d*, *q*) models for forecasting the disease burden of LOC during 2022–2031 was guided by criteria including the Akaike information criterion (AIC) and the Bayesian information criterion (BIC). Furthermore, the Ljung–Box *Q* test was conducted to verify that the residuals from the chosen models conformed to an independently distributed normal pattern.

The data analyses and visualizations were generated using the R statistical software program (version 4.2.0).

## Results

### Mortality, incidence, and prevalence

**Figure 1:**
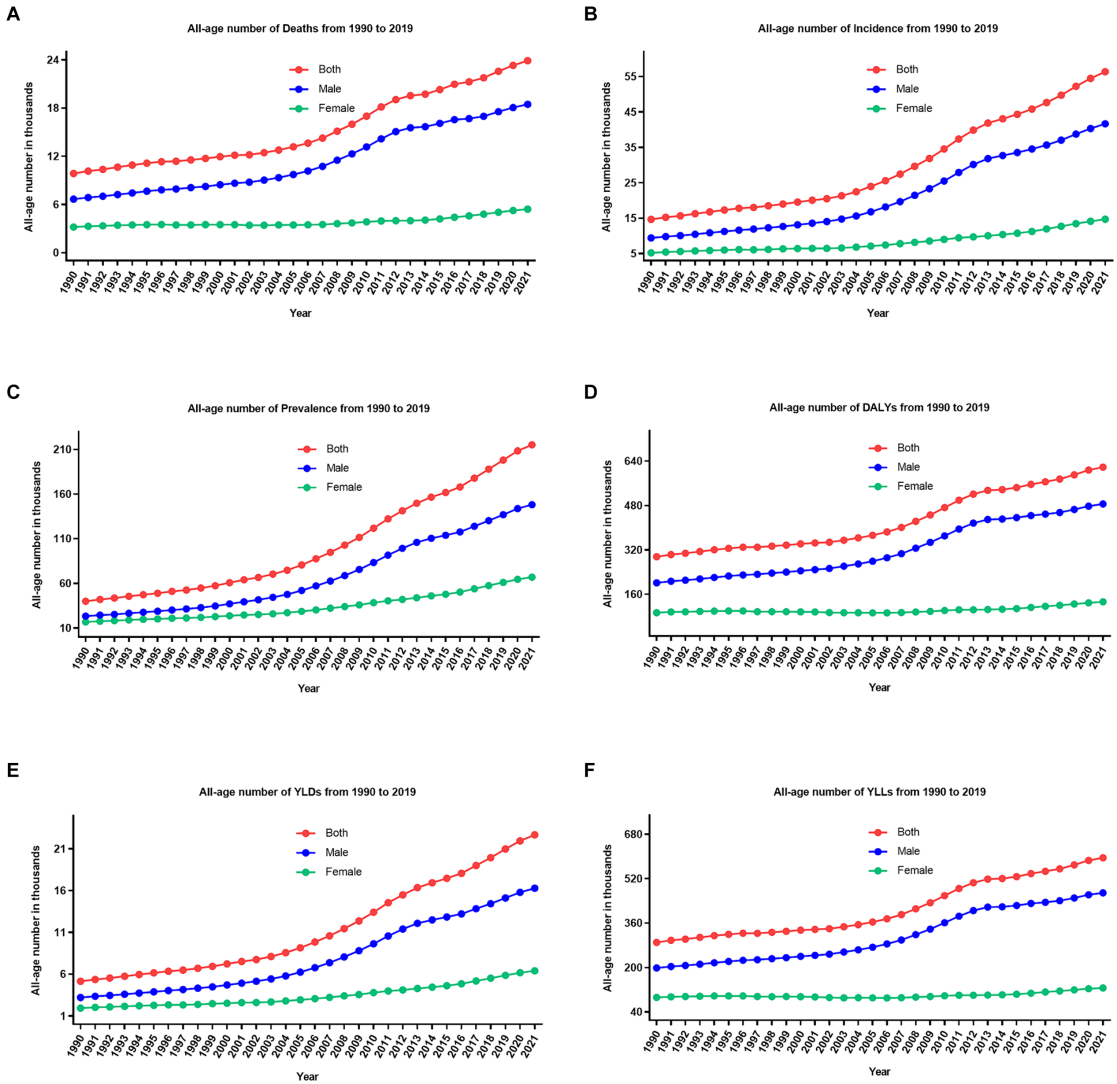
The trend of all-age number of all six measures for lip and oral cavity cancer in China from 1990 to 2021. **(A)** Deaths; **(B)** incidence; **(C)** prevalence; **(D)** DALYs; **(E)** YLDs; **(F)** YLLs.

**Figure 2:**
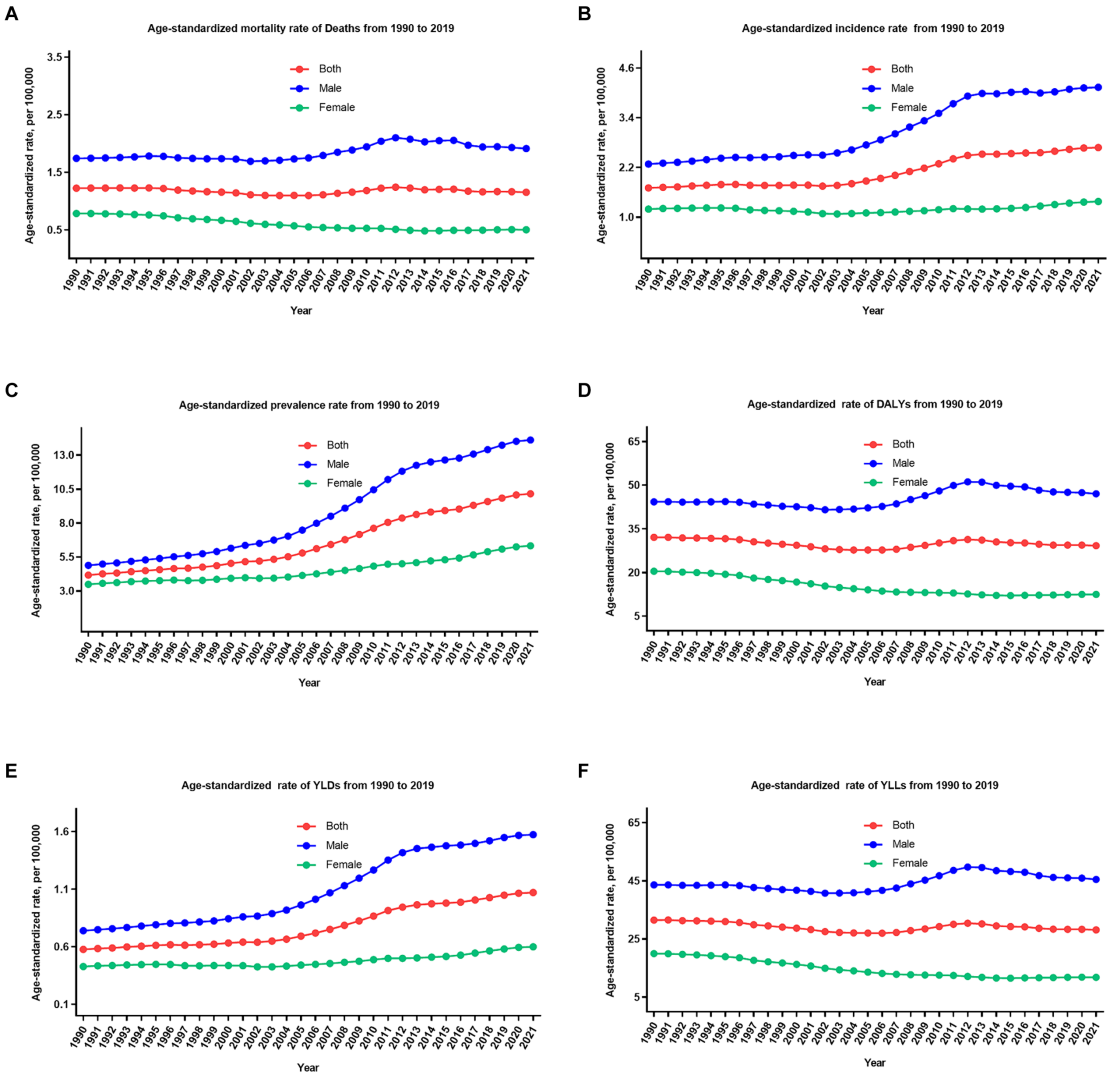
The trend of age-standardized rate of all six measures for lip and oral cavity cancer in China from 1990 to 2021. **(A)** Mortality rate; **(B)** incidence rate; **(C)** prevalence rate; **(D)** DALYs rate; **(E)** YLDs rate; **(F)** YLLs rate.

**Figure 3:**
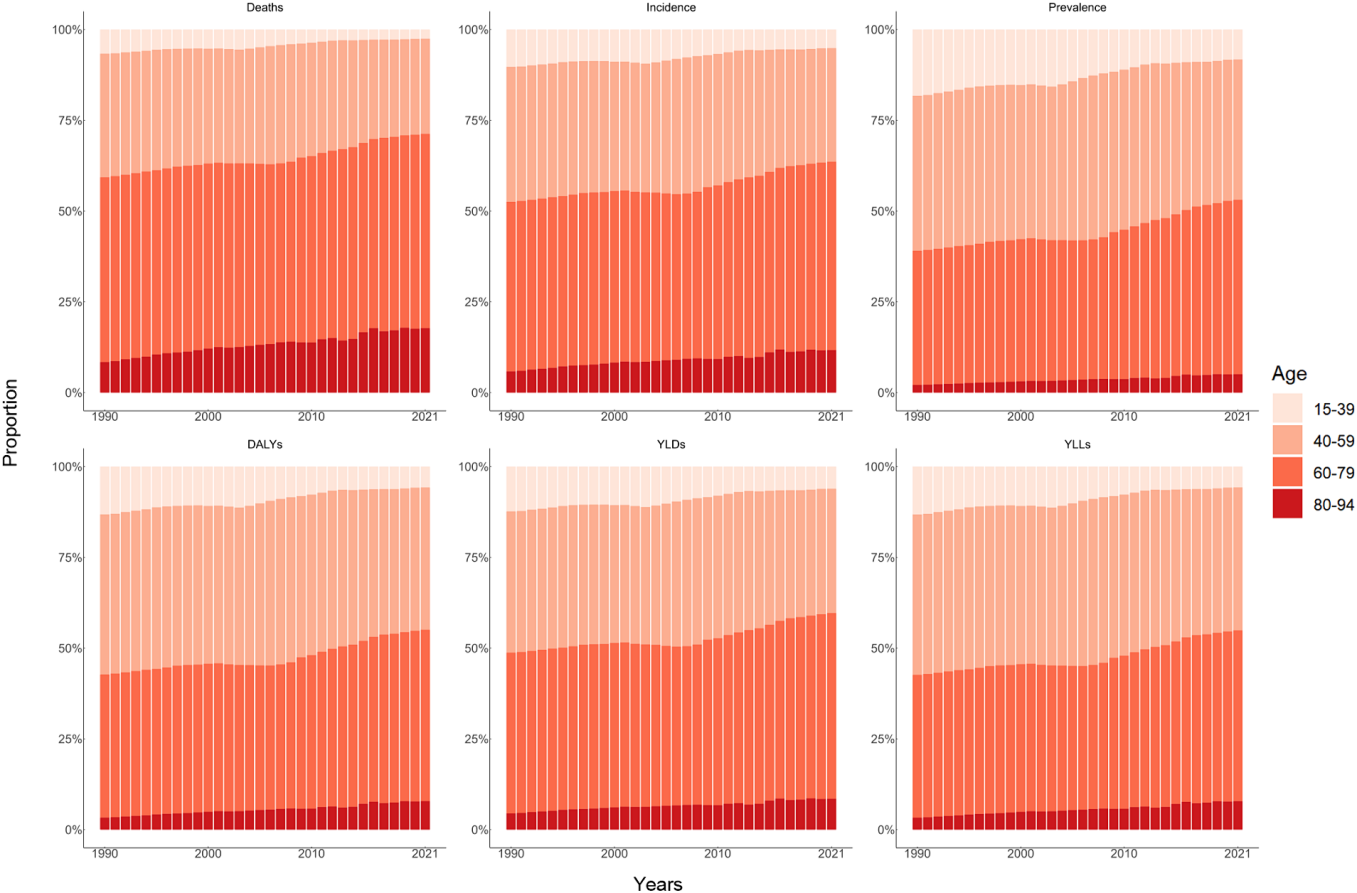
Age distribution of all-age number of six measures for lip and oral cavity cancer in China from 1990 to 2021.

The overall number and age-standardized rate of LOC and percentage changes by gender in China between 1990 and 2021 were presented in Table 1 and Figures 1A–C, 2A–C. Over the past 32 years studied here, deaths, incidence, and prevalence increased: death by 142.2% (81.1–219.8%), incidence by 283.7% (184.4–405.2%), and prevalence by 438.0% (301.9–601.7%). In 1990, the estimated numbers of deaths, incidence, and prevalence in China for LOC were 9.8 thousand (8.3–11.3), 14.6 thousand (12.3–16.9), and 39.9 thousand (33.6–46.3), respectively. In 2021, the values of the above three measures were 23.8 thousand (18.9–29.6), 41.6 thousand (31.1–54.2), and 215.1 thousand (172.1–264.2). Regarding gender stratification, males consistently exhibited higher numbers of deaths, incidence, and prevalence compared to females. Figure 3 illustrates the temporal trends in the number of LOC burden by age group. Overall, the majority numbers of all-age deaths, incidence and prevalence of LOC were recorded among those individuals aged 40–79 years. Concurrently, the age distribution of deaths, incidence and prevalence of LOC was relatively stable in China between 1990 and 2021, but there was an emerging transition of incidences from the young population (15–39 years) to the middle-aged and old population (40–94 years).

**Table 1:** All-age number and age-standardized rate of all measures for lip and oral cavity cancer and percentage changes by gender in China, 1990 and 2021.

Measures	All-age number in thousands (95% UI)			Age-standardized rate, per 100,000 (95% UI)		
	1990	2021	Change, %	1990	2021	Change, %
Deaths						
Total	9.8 (8.3, 11.3)	23.8 (18.9, 29.6)	142.2 (81.1, 219.8)	1.2 (1.0, 1.4)	1.1 (0.9, 1.4)	−5.8 (−28.6, 21.9)
Male	6.6 (5.4, 8.0)	18.4 (13.7, 23.8)	177.0 (88.1, 284.7)	1.7 (1.4, 2.0)	1.9 (1.4, 2.4)	9.8 (−23.3, 51.3)
Female	3.1 (2.6, 3.8)	5.4 (4.2, 6.7)	69.6 (25.5, 123.9)	0.7 (0.6, 0.9)	0.5 (0.3, 0.6)	−35.7 (−52.0, −15.3)
Incidence						
Total	14.6 (12.3, 16.9)	41.6 (31.1, 54.2)	283.7 (184.4, 405.2)	1.7 (1.4, 1.9)	2.6 (2.1, 3.3)	57.3 (18.6, 104.2)
Male	9.4 (7.7, 11.3)	41.6 (31.1, 54.2)	341.2 (196.6, 510.6)	2.2 (1.8, 2.7)	4.1 (3.1, 5.3)	81.0 (24.3, 149.4)
Female	5.2 (4.2, 6.3)	14.7 (11.4, 18.4)	180.3 (105.7, 278.3)	1.1 (0.9, 1.4)	1.3 (1.0, 1.7)	15.2 (−14.9, 55.3)
Prevalence						
Total	39.9 (33.6, 46.3)	215.1 (172.1, 264.2)	438.0 (301.9, 601.7)	4.1 (3.5, 4.8)	10.1 (8.1, 12.4)	143.7 (82.7, 217.0)
Male	23.2 (18.9, 27.9)	148.1 (110.2, 194.5)	538.2 (327.7, 788.4)	4.8 (3.9, 5.8)	14.1 (10.6, 18.4)	189.0 (95.3, 302.9)
Female	16.7 (13.6, 20.3)	66.9 (51.2, 84.9)	299.3 (192.8, 444.8)	3.4 (2.8, 4.2)	6.3 (4.8, 8.0)	81.6 (33.3, 147.6)
DALYs						
Total	295.3 (247.6, 342.3)	618.0 (487.5, 777.1)	109.2 (54.5, 178.7)	32.0 (27.0, 37.0)	29.2 (23.1, 36.4)	−8.9 (−32.3, 20.2)
Male	201.3 (163.6, 244.2)	485.2 (359.7, 634.3)	141.0 (61.1, 238.1)	44.3 (36.3, 53.4)	47.0 (35.1, 60.9)	6.2 (−28.0, 48.7)
Female	94.0 (76.1, 114)	132.7 (102.6, 167.2)	41.1 (3.2, 91.7)	20.4 (16.6, 24.6)	12.4 (9.6, 15.7)	−39.0 (−55.0, −17.4)
YLDs						
Total	5.1 (3.5, 6.9)	22.6 (15.3, 30.9)	341.2 (226.8, 486.7)	0.5 (0.3, 0.7)	1.0 (0.7, 1.4)	85.8 (38.7, 146.1)
Male	3.2 (2.1, 4.4)	16.2 (10.5, 22.9)	407.9 (244.4, 609.5)	0.7 (0.5, 1.0)	1.5 (1.0, 2.1)	113.1 (45.4, 200.0)
Female	1.9 (1.3, 2.6)	6.3 (4.2, 9.0)	230.7 (142.2, 352)	0.4 (0.2, 0.5)	0.5 (0.3, 0.8)	40.1 (2.7, 92.3)
YLLs						
Total	290.2 (244.0, 335.9)	595.3 (468.7, 752.8)	105.1 (51.7, 174.2)	31.5 (26.6, 36.2)	28.1 (22.2, 35.3)	−10.7 (−33.4, 18.4)
Male	198.1 (161.4, 239.6)	469.0 (347.6, 611.8)	136.7 (58.3, 232.6)	43.5 (35.8, 52.3)	45.4 (33.9, 58.8)	4.3 (−29.1, 46.3)
Female	92.1 (74.7, 111.8)	126.3 (97.3, 158.3)	37.1 (0.3, 85.8)	19.9 (16.3, 24.1)	11.8 (9.1, 14.8)	−40.6 (−56.3, −19.8)

UI, Uncertainty interval; DALYs, Disability-adjusted life years; YLDs, Years lived with disability; and YLLs, Years of life lost.

Following age standardization, mortality, incidence, and prevalence rates exhibited varied changes. The age-standardized mortality rate decreased by 5.8% (−21.9 to 28.6%), incidence and prevalence rate increased by 57.3% (18.6–104.2%), and 143.7% (82.7–217.0%) during these 32 years, from 1.2 per 100,000 (1.0–1.4) to 1.1 per 100,000 (0.9–1.4), 1.7 per 100,000 (1.4–1.9) to 2.6 per 100,000 (2.1–3.3), and 4.1 per 100,000 (3.5–4.8) to 10.1 per 100,000 (8.1–12.4), respectively. It is worth noting that the age-standardized mortality rate decreased by 35.7% (15.3–52.0%) for females and increased by 9.8% (−23.3 to 51.3%) for males during these 32 years. Furthermore, it is evident that the age-standardized incidence, mortality, and prevalence rates of LOC have demonstrated relatively stable fluctuations in recent years (since 2011).

### DALYs, YLDs, and YLLs

From 1990 to 2021, the number of all-age DALYs, YLDs, and YLLs of LOC increased by 109.2% (54.5–178.7%), 341.2% (226.8–486.7%), and 105.1% (51.7–174.2%), respectively (Table 1; Figures 1D–F, 2D–F). Additionally, a similar pattern of age distribution of all-age number of DALYs, YLDs, and YLLs of LOC can be observed in Figure 3. The age-standardized YLDs rates increased by 85.8% (38.7–146.1%) during these 32 years, from 0.5 per 100,000 (0.3–0.7) in 1990 to 1.0 per 100,000 (0.7–1.4) in 2021. Nevertheless, the age-standardized DALYs and YLLs rates decreased by 8.9% (−20.2 to 32.3%) and 10.7% (−18.4 to 33.4%) during these 32 years, from 32.0 per 100,000 (27.0–37.0) and 31.5 per 100,000 (26.6–36.2) in 1990 to 29.2 per 100,000 (23.1–36.4) and 28.1 per 100,000 (22.2–35.3) in 2021, respectively. During the past 32 years, there has been a consistent pattern indicating higher counts and rates of DALYs, YLDs, and YLLs among males compared to females. Particularly, the rate of DALYs and YLLs increased by 6.2% (−28.0 to 48.7%) and 4.3% (−29.1 to 46.3%) for males, respectively, decreased by 39.0% (17.4–55.0%) and 40.6% (19.8–56.3%) for females, respectively.

### Prediction of age-standardized rate by ARIMA models

In this research, the data and time series underwent transformation and differencing to achieve stable sequences, as evidenced by the results of the ADF test. The optimal parameters for the ARIMA models, along with their corresponding AIC and BIC values, were delineated in Supplementary Table S1. None of the residuals from the models exhibited significance upon undergoing testing, suggesting a good fit of the selected models to the data. From 2022 to 2031, the age-standardized rate of incidence, prevalence, and YLDs of LOC showed upward trends; while mortality, DALYs, and YLLs showed downward trends, and their estimated values were predicted to change to 2.72 per 100,000 (2.12, 3.31), 10.47 per 100,000 (7.34–13.61), 1.11 per 100,000 (0.80–1.41), 1.10 per 100,000 (0.72–1.48), 28.52 per 100,000 (23.45–33.60), and 27.43 per 100,000 (22.44–32.42) in 2031, respectively (Table 2).

**Table 2:** Prediction of age-standardized rate (per 100,000) of all six measures for lip and oral cavity cancer for the next 10 years according to ARIMA models with 95% confidence interval in China.

Measures	2022	2023	2024	2025	2026	2027	2028	2029	2030	2031
Deaths										
Both	1.15 (1.12, 1.17)	1.14 (1.08, 1.20)	1.14 (1.05, 1.23)	1.13 (1.01, 1.25)	1.13 (0.97, 1.28)	1.12 (0.93, 1.32)	1.12 (0.88, 1.36)	1.11 (0.83, 1.39)	1.11 (0.78, 1.44)	1.10 (0.72, 1.48)
Male	1.90 (1.83, 1.96)	1.88 (1.76, 2.00)	1.86 (1.69, 2.04)	1.85 (1.60, 2.10)	1.83 (1.51, 2.16)	1.82 (1.41, 2.22)	1.80 (1.31, 2.29)	1.79 (1.20, 2.37)	1.77 (1.09, 2.45)	1.75 (0.97, 2.54)
Female	0.50 (0.49, 0.52)	0.50 (0.46, 0.53)	0.50 (0.44, 0.55)	0.49 (0.41, 0.58)	0.49 (0.38, 0.61)	0.49 (0.34, 0.64)	0.49 (0.31, 0.67)	0.49 (0.27, 0.71)	0.48 (0.22, 0.74)	0.48 (0.18, 0.79)
Incidence										
Both	2.69 (2.65, 2.72)	2.69 (2.60, 2.79)	2.70 (2.55, 2.85)	2.70 (2.49, 2.92)	2.71 (2.42, 2.99)	2.71 (2.36, 3.06)	2.71 (2.30, 3.12)	2.71 (2.24, 3.19)	2.71 (2.18, 3.25)	2.72 (2.12, 3.31)
Male	4.15 (4.09, 4.22)	4.19 (4.02, 4.36)	4.23 (3.96, 4.50)	4.28 (3.91, 4.65)	4.33 (3.87, 4.79)	4.38 (3.84, 4.92)	4.43 (3.82, 5.05)	4.48 (3.80, 5.17)	4.54 (3.79, 5.29)	4.59 (3.78, 5.40)
Female	1.39 (1.37, 1.42)	1.40 (1.35, 1.46)	1.40 (1.32, 1.51)	1.43 (1.29, 1.57)	1.44 (1.26, 1.63)	1.46 (1.22, 1.69)	1.47 (1.17, 1.76)	1.48 (1.13, 1.84)	1.49 (1.07, 1.91)	1.51 (1.02, 1.99)
Prevalence										
Both	10.19 (10.07, 10.31)	10.22 (9.91, 10.53)	10.25 (9.70, 10.80)	10.28 (9.46, 11.11)	10.32 (9.17, 11.46)	10.35 (8.86, 11.83)	10.38 (8.52, 12.24)	10.41 (8.15, 12.67)	10.44 (7.75, 13.13)	10.47 (7.34, 13.61)
Male	14.22 (14.04, 14.39)	14.31 (13.92, 14.71)	14.41 (13.75, 15.07)	14.50 (13.54, 15.47)	14.60 (13.29, 15.90)	14.69 (13.01, 16.37)	14.79 (12.70, 16.87)	14.88 (12.37, 17.40)	14.98 (12.00, 17.95)	15.07 (11.62, 18.53)
Female	6.41 (6.31, 6.51)	6.49 (6.26, 6.71)	6.56 (6.19, 6.94)	6.64 (6.10, 7.19)	6.72 (5.98, 7.46)	6.80 (5.85, 7.75)	6.88 (5.70, 8.06)	6.96 (5.53, 8.38)	7.03 (5.35, 8.72)	7.11 (5.15, 9.07)
DALYs										
Both	29.04 (28.54, 29.54)	28.91 (27.88, 29.93)	28.81 (27.24, 30.38)	28.73 (26.61, 30.86)	28.67 (26.00, 31.34)	28.63 (25.43, 31.82)	28.59 (24.89, 32.29)	28.56 (24.39, 32.74)	28.54 (23.90, 33.18)	28.52 (23.45, 33.60)
Male	46.65 (45.89, 47.40)	46.24 (44.53, 48.29)	46.24 (43.32, 49.15)	46.12 (42.20, 50.04)	46.04 (41.18, 50.90)	45.98 (40.24, 51.73)	45.95 (39.38, 52.52)	45.92 (38.59, 53.26)	45.90 (37.85, 53.96)	45.89 (37.16, 54.62)
Female	12.43 (12.12, 12.74)	12.44 (11.75, 13.13)	12.44 (11.36, 13.53)	12.44 (10.91, 13.97)	12.44 (10.42, 14.47)	12.44 (9.87, 15.01)	12.44 (9.29, 15.59)	12.44 (8.67, 16.21)	12.44 (8.02, 16.87)	12.44 (7.33, 17.55)
YLDs										
Both	1.07 (1.06, 1.08)	1.07 (1.04, 1.10)	1.08 (1.02, 1.13)	1.08 (1.00, 1.17)	1.09 (0.97, 1.20)	1.09 (0.94, 1.24)	1.10 (0.91, 1.28)	1.10 (0.88, 1.32)	1.10 (0.84, 1.37)	1.11 (0.80, 1.41)
Male	1.58 (1.56, 1.60)	1.59 (1.54, 1.63)	1.59 (1.52, 1.67)	1.60 (1.49, 1.70)	1.60 (1.46, 1.74)	1.60 (1.43, 1.78)	1.61 (1.39, 1.82)	1.61 (1.36, 1.86)	1.62 (1.32, 1.91)	1.62 (1.29, 1.95)
Female	0.60 (0.59, 0.61)	0.61 (0.59, 0.63)	0.62 (0.58, 0.65)	0.62 (0.56, 0.68)	0.63 (0.55, 0.70)	0.63 (0.53, 0.73)	0.64 (0.51, 0.76)	0.64 (0.49, 0.79)	0.65 (0.47, 0.83)	0.65 (0.44, 0.86)
YLLs										
Both	27.96 (27.47, 28.46)	27.83 (26.82, 28.84)	27.73 (26.18, 29.28)	27.65 (25.56, 29.74)	27.59 (24.96, 30.21)	27.54 (24.40, 30.68)	27.50 (23.87, 31.14)	27.47 (23.37, 31.58)	27.45 (22.89, 32.01)	27.43 (22.44, 32.42)
Male	45.03 (44.30, 45.77)	44.80 (42.93, 46.68)	44.63 (41.75, 47.52)	44.52 (40.69, 48.34)	44.43 (39.72, 49.14)	44.37 (38.82, 49.92)	44.33 (38.00, 50.65)	44.30 (37.24, 51.35)	44.27 (36.53, 52.02)	44.26 (35.87, 52.65)
Female	11.82 (11.51, 12.13)	11.79 (11.10, 12.48)	11.76 (10.60, 12.92)	11.73 (10.03, 13.43)	11.69 (9.39, 14.00)	11.66 (8.70, 14.62)	11.63 (7.96, 15.30)	11.60 (7.17, 16.03)	11.57 (6.33, 16.80)	11.53 (5.45, 17.62)

DALYs, Disability-adjusted life years; YLDs, Years lived with disability; YLLs, Years of life lost.

### Attributable burden by selected risk factors

Attributable number and attributable age-standardized rate of mortalities, DALYs, YLDs, and YLLs were calculated for different gender groups in 2021, focusing on the two risk factors of tobacco and high alcohol use, as illustrated in Table 3. The attributable number of deaths for LOC including all risk factors was 13.4 thousand (9.8–17.9). For DALYs, it was 354.2 thousand (255.0–474.9). For YLDs and YLLs, they were 12.1 thousand (7.9–17.6), and 342.0 thousand (246.7–462.0), respectively. The attributable age-standardized rates of mortalities, DALYs, YLDs, and YLLs were 0.6 (0.4–0.8), 16.3 (11.8–21.8), 0.5 (0.3–0.8), and 15.8 (11.4–21.3) per 100,000 in 2021, respectively. Among the risk factors examined, tobacco and high alcohol use emerged as the predominant contributors to the burden of LOC, as indicated by both the attributable number and attributable age-standardized rates (Table 3; Figure 3). These results for males were higher than females.

**Table 3:** Points estimated and 95% uncertainty interval of attributable number and age-standardized rate of risk factors for lip and oral cavity cancer by gender in China, 2021.

Measures	All risk factors	Tobacco	High alcohol use
**Attributable number in thousands**			
Deaths			
Both	13.4 (9.8, 17.9)	10.3 (7, 14.2)	6.3 (4.4, 8.7)
Female	0.7 (0.5, 1.0)	0.5 (0.3, 0.7)	0.2 (0.1, 0.3)
Male	12.7 (9.1, 17.1)	9.8 (6.6, 13.6)	6.1 (4.3, 8.4)
DALYs			
Both	354.2 (255, 474.9)	266.6 (180.1, 370.5)	178.0 (125.0, 242.7)
Female	17.2 (12.1, 23.7)	12.3 (8.2, 17.6)	5.3 (3.3, 8.0)
Male	336.9 (239.8, 456.2)	254.3 (171.6, 356.8)	172.6 (121.4, 235.5)
YLDs			
Both	12.1 (7.9, 17.6)	9.1 (5.7, 13.8)	6.0 (3.7, 9.0)
Female	0.8 (0.5, 1.2)	0.5 (0.3, 0.9)	0.2 (0.1, 0.4)
Male	11.2 (7.2, 16.5)	8.5 (5.2, 13)	5.7 (3.6, 8.6)
YLLs			
Both	342.0 (246.7, 462.0)	257.5 (173.8, 357.8)	172 (121.2, 235.1)
Female	16.4 (11.5, 22.6)	11.7 (7.8, 16.8)	5.1 (3.1, 7.7)
Male	325.6 (232.8, 442.7)	245.7 (165.4, 344.4)	166.9 (117.7, 229.1)
**Attributable age-standardized rate per 100,000**			
Deaths			
Both	0.6 (0.4, 0.8)	0.4 (0.3, 0.6)	0.2 (0.2, 0.4)
Female	0.1 (0.1, 0.1)	0.1 (0.1, 0.1)	0.1 (0.1, 0.1)
Male	1.2 (0.9, 1.7)	0.9 (0.6, 1.3)	0.6 (0.4, 0.8)
DALYs			
Both	16.3 (11.8, 21.8)	12.1 (8.2, 16.8)	8.3 (5.8, 11.3)
Female	1.5 (1.1, 2.1)	1.1 (0.7, 1.5)	0.4 (0.3, 0.7)
Male	32.0 (23.0, 43.1)	23.9 (16.1, 33.5)	16.4 (11.6, 22.3)
YLDs			
Both	0.5 (0.3, 0.8)	0.4 (0.2, 0.6)	0.2 (0.1, 0.4)
Female	0.1 (0.1, 0.1)	0.1 (0.1, 0.1)	0.1 (0.1, 0.1)
Male	1.0 (0.6, 1.5)	0.8 (0.4, 1.2)	0.5 (0.3, 0.8)
YLLs			
Both	15.8 (11.4, 21.3)	11.7 (7.9, 16.2)	8.0 (5.7, 10.9)
Female	1.4 (1.0, 2.0)	1.0 (0.7, 1.5)	0.4 (0.2, 0.7)
Male	31.0 (22.3, 41.9)	23.1 (15.6, 32.3)	15.9 (11.2, 21.7)

DALYs, Disability-adjusted life years; YLDs, Years lived with disability; YLLs, Years of life lost.

## Discussion

This nationwide epidemiological study, conducted at the population level, delivers contemporary insights into the disease burden of LOC in China from 1990 to 2021. We observed a significant increase in the disability burden, coupled with a decrease in the death burden attributed to LOC within this timeframe. Furthermore, our study predicts the continuation of these trends over the upcoming decade in China. The analysis also underlines the critical role of tobacco and high alcohol use as principal risk factors for LOC.

The frameworks of LOC burden have been substantial shifted in China, diverging significantly from global, as well as other regional and national trends, observed over the past three decades (2, 7, 19, 20). The total number of LOC burden (all measures) increased over the study period in China, driven by rapid population growth. It is widely recognized that age and the aging process play pivotal roles in the onset and progression of LOC, with older demographics often encountering diminishing health capacities and heightened functional constraints (21). Notably, China’s older population proportion continues to ascend, juxtaposed against a declining birth rate, a trend expected to persist in the foreseeable future (22). As China’s demographic landscape continues to tilt toward aging, an unsustainable demographic structure could emerge as a significant contributing factor to the escalating burden of LOC. Meanwhile, a worrisome revelation indicates that even after standardizing for age effects, the impact of LOC conditions on China remains substantial over the observed period. Previous global studies have shown regions with high SDI displaying a decreasing trend in age-standardized mortality, YLL, and DALY rates from 1990 to 2019 (2, 19), consistent with our findings. It is imperative to acknowledge China’s commendable strides in combating the burden of cancers over the past 3 decades. These achievements encompass an expansion in the dental practitioner workforce (23), the issuance of pertinent policy directives (24), and ongoing innovations in immunotherapy regimens and surgical management (25, 26). However, despite these efforts, the current allocation of medical resources and intervention strategies appears inadequate in curbing the escalating disability burden of LOC in China. Looking ahead, While the incidence and prevalence rates are expected to rise, the concurrent decline in mortality, YLLs and DALYs offers a glimmer of hope, suggesting that current healthcare strategies might be effective in mitigating the lethal aspects of the disease. Nonetheless, the projected increase in YLDs necessitates focused attention on palliative care and quality-of-life interventions.

More importantly, our results also suggest that premature death is the primary contributor to the burden of LOC in China, given that pain, swallowing difficulties, and eating challenges may result in devastating consequences for patients with LOC. The mortality rate of LOC is notably high, contingent upon the disease stage at diagnosis (27). Delayed diagnosis is common in China, as there is a tendency to prioritize diseases that are fatal and manifest with evident symptoms, coupled with a lack of understanding of malignant oral tumors and limited access to primary healthcare, particularly in economically disadvantaged regions. Upon diagnosis of LOC, nearly half of the patients exhibit advanced symptoms, leading to a significant decline in quality of life and premature death (28), underscoring the importance of enhancing public awareness of oral health and strengthening early diagnostic techniques as key avenues for mitigating the burden of LOC, as well as the critical need to pay closer attention to the death burden.

**Figure 4:**
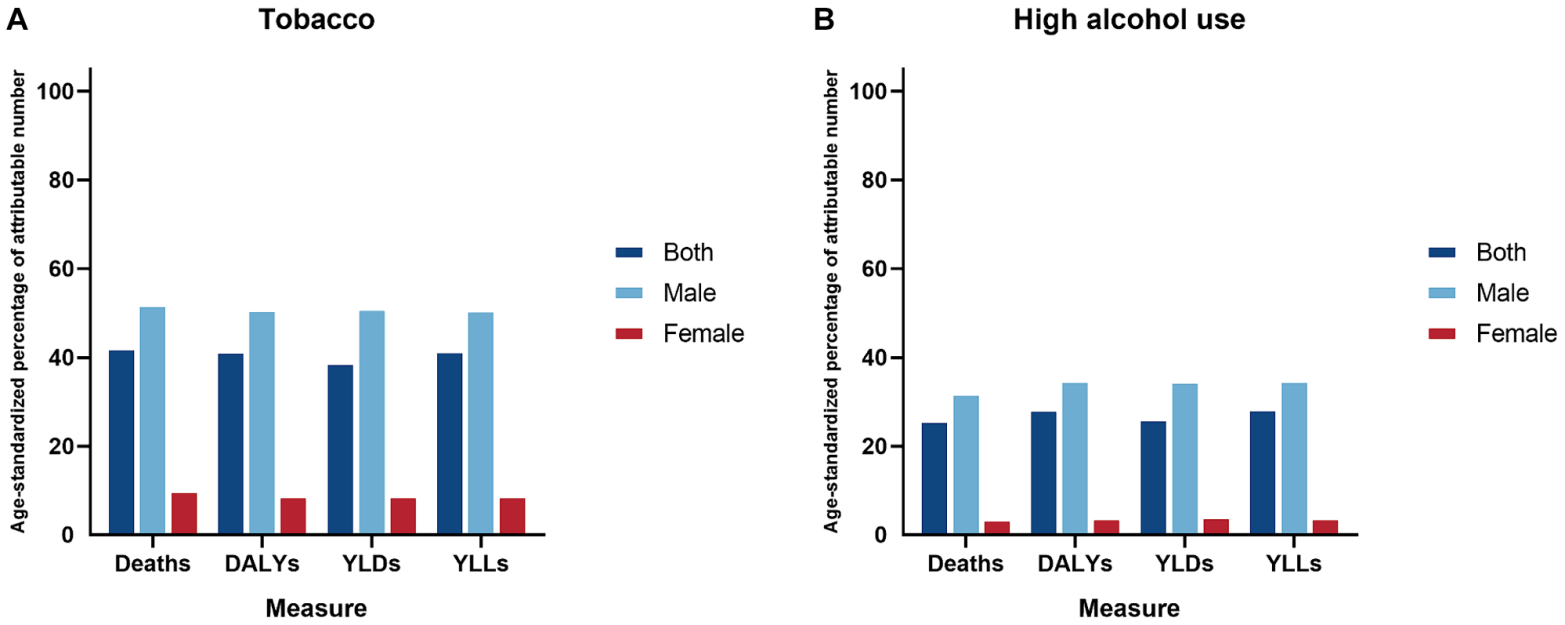
Age-standardized percentage of attributable number two risk factors for lip and oral cavity cancer by gender in China, 2021. **(A)** Tobacco; **(B)** High alcohol use.

The present findings of high PAFs (Figure 4) highlight the importance of addressing risk factors such as tobacco and high alcohol use, as key approaches to reduce LOC burden. The etiological significance of tobacco and high alcohol use in the pathogenesis of LOC cancer is underscored by extensive molecular and epidemiological studies. Tobacco smoke, a complex mixture of over 6,000 compounds, contains numerous carcinogens, which induce genetic mutations and disrupt cellular homeostasis, thereby promoting malignant transformation (29). These carcinogens interact with DNA, leading to the formation of DNA adducts, which if not adequately repaired, can result in mutations that drive cancer progression (29). High alcohol use, particularly in the form of ethanol, is metabolized to acetaldehyde, a known carcinogen that induces DNA damage and inhibits DNA repair mechanisms (30). Acetaldehyde also enhances the carcinogenic effects of tobacco by facilitating the absorption of tobacco-derived carcinogens and increasing the production of reactive oxygen species, which contributes to oxidative stress and further DNA damage (31). As the world’s largest producer and consumer of tobacco, China experiences a high prevalence of smoking and significant disease burden (32). Active smoking, widely accepted as a behavior, particularly prevalent among males, is believed to stimulate social interaction and alleviate stress (33). Furthermore, in contrast to smoking, chewing tobacco is associated with comparatively fewer deaths and DALYs (34). Nevertheless, caution should still be exercised regarding the future burden of diseases caused by chewing tobacco. The alarming reality of the high disability and death burden caused by tobacco in China in 2021 underscores once again the importance of strengthening the implementation of tobacco control measures, despite ongoing efforts in the anti-smoking campaign. Alcohol has a long history in China, and in modern Chinese society, drinking remains a cultural norm (35). The rapid economic and cultural changes have facilitated the growth of the alcohol market in China (36). Hazardous drinking behaviors, exemplified by excessive or recurrent alcohol consumption, are escalating to epidemic proportions within the demographic landscape of China (37). Furthermore, the exposure to tobacco and alcohol significantly varies across regions in China, influenced by cultural customs, socioeconomic status, and policy frameworks (38). In particular, the central and western regions report higher prevalence rates of tobacco use and alcohol consumption, correlating with increased risks of LOC (38, 39). This variability in risk factor exposure necessitates a region-specific approach in risk assessment and in the design of intervention strategies. To alleviate the impact of these risk factors, a comprehensive, multi-component strategy is essential. In areas with substantial exposure to tobacco and alcohol, community-focused interventions, such as smoking cessation programs and alcohol abuse prevention, are critical. Educational initiatives should emphasize the health hazards linked to these practices, and the availability of cessation services should be expanded (39). Moreover, implementing policies like taxation on tobacco and alcohol, coupled with stringent regulations governing the sale and marketing of these substances, has proven efficacious in curbing their consumption (32, 35).

Lip and oral cavity cancer may exhibit inconsistent impacts on males and females in China, with a predictably higher burden among males, possibly due to prevalent risk exposures (22). The present study shows that the PAFs of tobacco and high alcohol use for LOC is much higher in men than in women, highlighting the focus on management of risk factors. Biological mechanisms such as differences in oral microbiome composition, hormonal influences, and immune responses may also contribute to the observed gender-specific differences (40, 41). Furthermore, females often place greater emphasis on their physiological well-being than males and may have better access to healthcare services and higher health-seeking behaviors, which could contribute to earlier detection and better outcomes (42), potentially prompting them to adopt control measures at an earlier stage. Age-specific patterns in the LOC burden reflect the cumulative exposure to carcinogens and the latency period of the disease. The majority of the LOC burden was recorded among individuals aged 40–79 years, and there was an emerging transition of numbers from the young population to the middle-aged and older population, which aligns with the prolonged duration of exposure to tobacco and high alcohol use. Moreover, the aging process itself contributes to increased susceptibility to carcinogenesis through mechanisms such as genomic instability, impaired DNA repair, and chronic inflammation (43).

This study provides timely insights into the incidence, prevalence, mortality, DALYs, YLDs, YLLs, and primary risk factors associated with LOC in China spanning from 1990 to 2021, while also offering reasonable predictions regarding disease burden over the next decade. The methodology employed is widely recognized within GBD studies and has demonstrated robustness. However, it is imperative to acknowledge several limitations. First, elucidating the precise determinants underlying regional and provincial differentials in disease burden remains a pivotal yet elusive endeavor, necessitating further exploration. Persistent health inequities and variances in healthcare accessibility among provinces underscore the imperative for evidence-driven health policy formulation and implementation at the provincial level throughout China. Second, our study, while utilizing the extensive GBD 2021 database, predominantly concentrates on the classical risk factors of tobacco and high alcohol use in relation to LOC. This limited scope fails to consider other potential etiological factors such as dietary habits, oral hygiene practices, and genetic predispositions. This narrow focus constitutes a significant limitation, underscoring the necessity for future research to incorporate a broader spectrum of risk factors to enhance our understanding of LOC in China. Third, the study does not address the effect sizes, heterogeneity, and sensitivity analyses of risk factors associated with LOC, due to its reliance on cross-sectional data from the GBD study. These analyses are crucial for discerning the relative contributions and robustness of various risk factors.

## Conclusion

Between 1990 and 2021, the disability burden from LOC in China increased, while the death burden decreased. Projections suggest these trends will persist over the next decade, suggesting that current healthcare strategies may be mitigating the fatal aspects of the disease. A key finding highlighted by our quantitative analyses is the attribution of a significant portion of this disease burden to modifiable risk factors, specifically tobacco use and excessive alcohol consumption, predominantly affecting males and individuals aged 40–79 years. Interventions should prioritize reducing these preventable exposures to strengthen tobacco and alcohol control measures. Tailored, population-specific strategies can effectively reduce the LOC disease burden across China.

## Data Availability

The datasets presented in this study can be found in online repositories. The names of the repository/repositories and accession number(s) can be found below: http://ghdx.healthdata.org/gbd-results-tool.

## Footnotes

^1^ https://ghdx.healthdata.org/gbd-2021
