# Supplementary material for: Disease burden and attributable risk factors of lip and oral cavity cancer in China from 1990 to 2021 and its prediction to 2031
Source: Front Public Health. 2024 Sep 5;12:1419428. doi: 10.3389/fpubh.2024.1419428 (PMC11413874; doi:10.3389/fpubh.2024.1419428)
Supplement: Supplementary file 1 [file Table_1.DOCX]

**Disease burden and attributable risk factors of** **lip and oral cavity cancer in China from 1990 to 2021 and its prediction to 2031**

**Supplementary material**

**Table S1** ARIMA model parameters and their corresponding AIC and BIC for prediction of age-standardized rate (per 100,000) of all six measures for lip and oral cavity cancer for the next 10 years in China

| **Measures** |  | | |
| --- | --- | --- | --- |
| Deaths | Parameters | AIC | BIC |
| Both | ARIMA(2,2,0) | -168.31 | -164.11 |
| Male | ARIMA(0,2,1) | -117.03 | -114.23 |
| Female | ARIMA(0,2,0) | -203.67 | -202.27 |
| Incidence |  |  |  |
| Both | ARIMA(1,1,1) | -153.77 | -149.47 |
| Male | ARIMA(1,1,1) | -113.96 | -108.22 |
| Female | ARIMA(0,2,0) | -174.94 | -173.54 |
| Prevalence |  |  |  |
| Both | ARIMA(0,2,1) | -79.71 | -76.91 |
| Male | ARIMA(0,2,0) | -57.40 | -56.00 |
| Female | ARIMA(0,2,0) | -91.51 | -90.11 |
| DALYs |  |  |  |
| Both | ARIMA(1,1,0) | 7.55 | 10.42 |
| Male | ARIMA(2,1,1) | 39.52 | 45.26 |
| Female | ARIMA(2,2,0) | -19.58 | -15.38 |
| YLDs |  |  |  |
| Both | ARIMA(2,2,2) | -209.44 | -202.43 |
| Male | ARIMA(1,1,0) | -187.15 | -184.29 |
| Female | ARIMA(1,2,1) | -227.66 | -223.45 |
| YLLs |  |  |  |
| Both | ARIMA(1,1,0) | 6.45 | 9.31 |
| Male | ARIMA(1,1,2) | 38.55 | 44.29 |
| Female | ARIMA(0,2,0) | -23.50 | -22.10 |
